# CO-Creation and Evaluation of Food Environments to Advance Community Health (COACH)

**DOI:** 10.1016/j.focus.2023.100111

**Published:** 2023-05-27

**Authors:** Jillian Whelan, Julie Brimblecombe, Meaghan Christian, Carmen Vargas, Megan Ferguson, Emma McMahon, Amanda Lee, Colin Bell, Tara Boelsen-Robinson, Miranda R. Blake, Meron Lewis, Laura Alston, Steven Allender

**Affiliations:** 1 Global Centre for Preventive Health and Nutrition (GLOBE), Institute of Health Transformation, School of Medicine, Deakin University, Geelong, Australia; 2 Department of Nutrition, Dietetics and Food, Faculty of Medicine, Nursing and Health Science, School of Clinical Sciences, Monash University, Melbourne, Australia; 3 Global Centre for Preventive Health and Nutrition (GLOBE), Institute of Health Transformation, School of Health and Social Development, Deakin University, Geelong, Australia; 4 School of Public Health, The University of Queensland, Brisbane, Australia; 5 Wellbeing and Preventable Chronic Disease Division, Menzies School of Health Research, Darwin, Australia; 6 Deakin Rural Health, School of Medicine, Deakin University, Warrnambool, Australia

**Keywords:** Food environments, cocreation, health promotion, frameworks, practical guidance, continuous quality improvement

## Abstract

**Introduction:**

Food environments are a key determinant of food intake and diet-related health. This paper describes the development of an iterative, adaptive, context-specific framework for health-enabling food environments embedded in cocreation theory.

**Methods:**

A 3-stage multimethod framework for the coproduction and prototyping of public health interventions was followed in an iterative manner during the development of the framework. These 3 stages were (1) evidence review, including systematic review, consultation with experts, and observation of current work; (2) codesign of the framework prototype with multiple stakeholders; and (3) coproduction through refinement of the prototype through stakeholder workshops and expert reviews with incorporation of researcher notes and workshop evaluation. We use the term prototype during the development phase and the term framework to report on the final product.

**Results:**

COACH (CO-creation and evaluation of food environments to Advance Community Health) is a process framework that describes what best practice application of cocreation in health-enabling food retail environments should involve. COACH consists of 10 interdependent factors within a 4-phase continuous quality improvement cycle. The 4 phases of the cycle are engagement and governance establishment, communication and policy alignment, codesign and implementation, and monitoring and evaluation.

**Conclusions:**

Utilizing cocreation theory represents an innovative step in research and practice to improve the healthiness of food retail environments. COACH provides a specific, unique, and comprehensive guide to the utilization of cocreation to improve the healthiness of food environments in practice.

## HIGHLIGHTS

• Existing food systems contribute to chronic disease.
• Co-creation theory can inform the creation of health-enabling food retail environments.
• Multiple stakeholders should be engaged at relevant stages of co-creation.
• A best practice co-creation framework is developed from research and practice.
• The framework (COACH) is supported by a checklist for each stage of change.

## INTRODUCTION

WHO's target to halt the global rise in diabetes, overweight, and obesity in adolescents and adults by 2020 has been extended to 2030 owing to a lack of progress^1^ and recognition attributed in part to the complexity of the drivers of obesity and pointing to the need for systems approaches to impact change.^2^ This global priority will require the achievement of healthy and sustainable diets^3^ and reduced reliance on ultraprocessed food.^4^ Unhealthy diets have been linked with food systems that provide limited healthy options and thereby contribute to chronic disease.^5^ These food systems result from the complex interplay between the production, processing, distribution, preparation, and consumption of food, and the outputs of these activities include socioeconomic and environmental factors.^6^ Typically, a food system comprises 3 broad elements: food supply chains (e.g., producers, logistics, retailers, and others), food retail environments (i.e., supermarkets, corner stores, cafes), and consumer behavior (purchasers).^7^ Food retail environments are where most food is accessed and can have a powerful impact on what customers purchase and subsequent health.

Although recent policies to improve the healthiness of food environments include taxes on ultraprocessed drinks in more than 45 countries, few countries have adopted taxes on ultraprocessed foods, and none have added major healthy food subsidies,^4^ although Australia has continued to exempt fresh food from their Goods and Services Tax.^8^ Benchmarking of the food retail environment has revealed poor performance on multiple international health-rating scales,^9^^,^^10^ leading to calls for action on national and international policy mandates such as food taxes, labelling, and reformulation.^11^ Several policy mandates have been implemented in other health priority areas, for example, restriction of tobacco sales through legislating a minimum age for purchasing cigarettes^12^ or taxes to reduce sugar sweetened beverage sales.^13^

However, given that food retail environments remain unhealthy,^14^, ^15^, ^16^ broader systemic innovations and complementary methods to improve the healthiness of food environments should be explored to improve this key determinant of population health.^17^^,^^18^ Food retail environments comprise diverse stakeholders, potentially holding competing priorities, within the multiple layers of the food environment. Therefore, an iterative approach may be required in food environment research. Using cocreation provides an avenue to build health-enabling food environments mutually beneficial to all stakeholders within the food supply.^19^ Cocreation involves all stakeholders (such as community, business, food suppliers, governments, and others) from exploration and articulation of needs to the creation, implementation, and evaluation of public health initiatives.^20^ Cocreation has shown potential to empower and enhance efforts to create change in diverse fields such as technology, business, design, and community development.^21^^,^^22^ Leask et al.^22^ proposed a checklist for reporting initiatives that utilized cocreation as a participatory action research method time. Their checklist considers cocreation as an event that occurs at a point in time rather than over a series of interactions at multiple time points, as is likely required when attempting to change food environments.

The potential of cocreation has been shown^23^ by studies such as the study by Brimblecombe et al.,^24^ who codesigned a food retail initiative across multiple remote Aboriginal and Torres Strait Islander communities and who, through the use of an RCT design, found mutually beneficial outcomes of reduced sales of unhealthy products without a reduction in profit. Bogomolova et al.^23^ codesigned a healthy choice supermarket program with consumers and retailers (product placement with supplementary education programs and media campaign), which increased knowledge of healthy options, but the impact on sales varied. Young et al.^25^ codesigned a shelf placement intervention for breakfast cereals. Together, these studies that utilize codesign with multiple stakeholders show that the use of codesign is feasible for engaging with the retail sector in changing food environments. The aim of this paper is to present the development and operationalization of a process framework that describes what best practice application of cocreation in health-enabling food retail environments should involve.

## METHODS

A 3-stage multimethod framework for the coproduction and prototyping of public health interventions^26^ was followed iteratively during the development of the framework. These 3 stages were (1) evidence review, including a systematic review, consultation with experts, and observation of current work; (2) codesign of the framework prototype with multiple stakeholders; and (3) coproduction through refinement of the prototype through stakeholder workshops and the incorporation of researcher notes and workshop evaluation. We use the term prototype to describe the development phase and the term framework to report on the final product. Ethical approval for all workshops involving stakeholders was granted by Deakin University's Human Research Ethics Committee (HEAG-H 165_2021), and all participants provided informed consent to be involved in the project.

### Recruitment

Participation in this study comprises 2 groups: first, academic experts who commenced the prototype development and were involved in ongoing evolution and improvement of the prototype through multiple iterations, and second, experts from health promotion practice who provided feedback on the prototype development and informed final framework. Academic experts were recruited by invitation through the Centre for Research Excellence (CRE) in food retail environments to promote health.^27^

Practice experts were recruited through the Nourish Network,^28^ Food Innovation Australia,^29^ the Australian Prevention Partnership Centre,^30^ and author's networks (ethics approval: HEAG-H 165_2021). Registration for the workshop participants was facilitated through the online Eventbrite events management platform.

**Figure 1:**
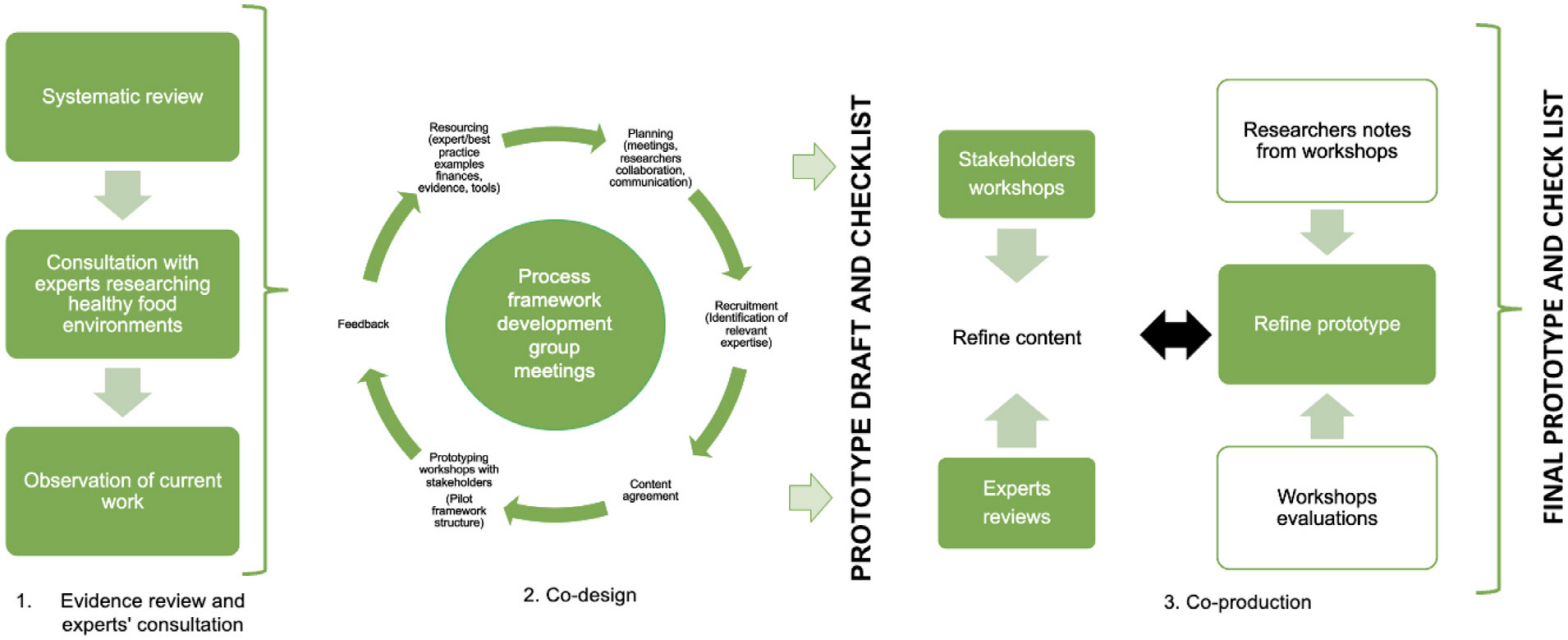
Stages of the multimethod development of a best practice prototype for the application of cocreation of health-enabling food retail, based on Hawkins et al.^26^

A summary of the methodology is shown in Figure 1, and Table 1 identifies the methods used at each step within the 3 stages. The visual depiction of the prototype and the framework were created using the STICKE software.^31^

**Figure 2:**
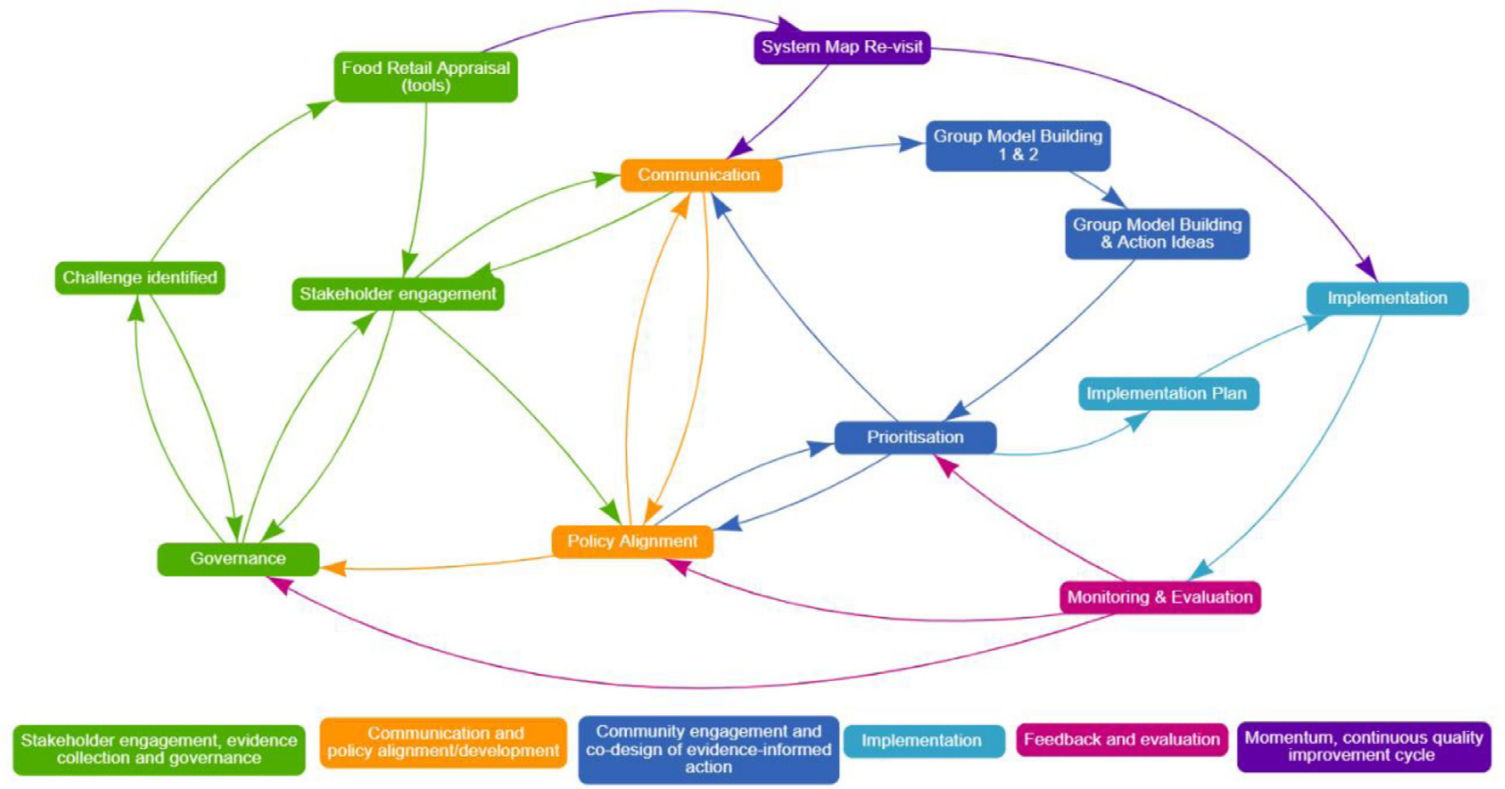
Prototype: COACH. COACH, CO-creation and evaluation of food environments to Advance Community Health.

**Table 1:** Overview of the Methods Used at Each Stage of the Prototype and Framework Development

Stage	Methods used
Stage 1: Evidence reviews and experts consultation	Systematic review
	Consultation with academics
	Observation of current work
Stage 2: Codesign	Resourcing (academic and best practice examples, finance, evidence, tools)
	Planning (meetings, academics, collaboration, communication)
	Recruitment (identification of relevant expertise)
	Content agreement
	Prototyping workshops with academics (draft framework structure)
	Feedback and refinement
Stage 3: Testing prototype with experts	Stakeholder workshops; 2 online workshops for 2.5 hours each
	On-line survey of stakeholder participants
	Academic review (final framework structure)
	Researcher notes and workshop evaluations

The refined prototype is presented (Figure 2).

**Table 2:** Overview of the Results or Outputs Created at Each Stage of the Prototype and Framework Development

Stage	Result/output
Stage 1: Evidence reviews and experts consultation	A systematic review of the use of cocreation in food retail environments to promote health (published).^19^
	Initial workshop of the authorship team to map priority steps in cocreation (1 workshop). Interviews with researchers who have published in the field of healthy food retail provided additional insights into the core elements of successful cocreation: *n*=9
	Existing publications that describe elements of cocreation of healthy food retail were mapped to the phases of the prototype for cocreation and shown in Appendix File 1 (available online). This work was completed over 2 × 1-hour meetings. Concepts in protocol development were drawn from multiple sources, particularly published work related to the Good Food Systems Project, where governance, communication, and the continuous quality improvement cycles of PLAN-DO-Collect–LEARN were included.^29^
Stage 2: Codesign	Methods, stages of cocreation, and relevant tools were extracted from best practice examples within 2 × 2-hour online meetings by authors: JW, JB, AL, MF, EM, LA, SA.^24^^,^^28^, ^29^, ^30^, ^31^, ^32^
	Across 2 further sessions, authors (JW, JB, EM, MF, AL, LA, CB) worked individually to identify how the methods, stages of cocreation, and relevant tools identified in the previous step could be envisaged as a nonlinear continuous process. After working individually, each participant explained to the broader research team how they each conceived that these elements fit together. This took place in an iterative manner over 3 meetings.
	Additional specific food retail expertise was identified within the international RE-FRESH collaboration partnership project
	The prototype was presented for feedback to a meeting of 12 researchers of the RE-FRESH collaboration.^27^
	The research group iteratively refined this draft process framework. Over the course of 3 more workshops, each of 1-hour duration, drawing upon existing literature (Appendix File 1, available online) and researcher field experience, the initial prototype was developed. A checklist for COACH was developed to guide users in the application of this prototype.
	The initial prototype was then refined on the basis of the feedback provided across Stage 2. This is shown in Figure 1.
Stage 3: Testing prototype with experts	This prototype was subsequently presented to 15 researchers (members of CRE RE-FRESH)^27^ and 44 community-based health promotion professionals through 2 workshops held in 2021. Within each of the 2 workshops, researchers presented (1) a description of the prototype and demonstration of the electronic tools that support elements of the framework and (2) a mock community (Coachville) to show the prototype in action to assist stakeholders (see Appendix File 2, available online, for the workshop outline).
	An all-authorship team workshop was held on March 18, 2022 (4-hour duration), and a further 4 academics attended for feedback. The prototype was reviewed, discussed, and updated to the framework proposed in this study. The checklist for COACH was refined to guide the application of the framework within research and practice.
	Researchers utilized iterative listening and content analysis to code and analyze audio recordings of the workshops.^33^ Quantitative survey data were analyzed using simple descriptive statistics (e.g., frequency and proportions to response options). This process led to improvements in the quality and use of the framework and COACH checklist. On the basis of this feedback, the protocol was further refined.
	See Figure 2

COACH, CO-creation and evaluation of food environments to Advance Community Health; RE-FRESH, Centre of Research Excellence in Food Retail Environments for Health.

## RESULTS

After the 3 stages of prototyping (Table 2), a process framework that describes what best practice application of cocreation in health-enabling food retail environments should involve was created. The CO-creation and evaluation of food environments to Advance Community Health (COACH) is presented to guide research and practice.

### Stage 1: Evidence Review and Expert Consultation

The systematic review identified 23 articles that utilized various aspects of cocreation to improve the healthiness of food retail environments.^19^ Cocreation of healthy food retail was most commonly used when developing initiatives with specific population groups, such as lower socioeconomic and indigenous communities where the needs and perspectives of these groups that are often marginalized in decision making may be overlooked.^32^, ^33^, ^34^, ^35^, ^36^ Key elements of cocreation were the type of stakeholders involved, their level of engagement, and their motivations.^19^

Interviews with 9 academic experts who have published in the field of codesign in healthy food retail identified 3 core constructs in cocreation: (1) stakeholder identification; (2) motivations and interactions of these stakeholders; and (3) barriers and enablers, which included resourcing, effective and trusting relationships, and open communication (Appendix File 1, available online).

### Stage 2: Codesign

The first prototype of COACH included 13 core constructs across 5 phases and sought to provide specific guidance on methods and tools that could be used in each stage and phase (Fig. 2). The prototype was illustrated through the use of STICKE software.^31^ A draft checklist for COACH was developed for guidance in the use of this prototype and further developed into the checklist shown in Table 3.

**Table 3:** COACH Checklist Provides Guidance on Factors to Consider When Working Through Each Phase of COACH

Checklist		
COACH phases	Component	Checklist for implementing COACH
Stakeholder engagement, evidence collection, and governance	Challenge identified	Has the key area of concern been identified by stakeholders?
	Governance and accountability	Is there an existing relevant stakeholder network? Are key stakeholders represented in this network? Is there clear and apparent strategic leadership within the governance group? Are there strategies for relationship building among the stakeholders? Is a formal memorandum of understanding or agreement required? Have terms of reference been set?
	Stakeholder engagement	Have all relevant community stakeholders been identified? Have the benefits and commitments of engagement with this initiative being fully communicated? Are there well-defined roles and responsibilities for the stakeholders? Have ethical approval, organizational consent, relevant privacy considerations been met as relevant? Have relevant secretarial functions been planned (e.g., meetings, resources)?
	Appraisal	Do you have access to appropriate valid and reliable tools to measure and assess change in the food environment? What are they and when will they be used? E.g., Store Scout App, Healthy diets ASAP tool Nutrition Environment Measure Survey https://nems-upenn.org/publications/
Communication policy alignment and development	Communication and information sharing	Are there clear communication mechanisms among the stakeholders? Are communication plans and strategies developed across multiple media as relevant to the particular stakeholders/communities?
	Policy and organizational policy	Is there a government policy that needs to be implemented that the initiative aligns with? Is there an organizational policy in existence or could one be developed to support implementation and sustainment? For example, Healthy Stores 2020 Policy Action Series: healthy Stores 2020 Policy Action series: healthy policy to support retailers and communities (healthyfoodretail.com) https://foodenvironmentdashboard.com.au/
Codesign of evidence-informed action and implementation planning	Codesign and prioritization of proposed actions	Has group model building or other co-design methodology, e.g., focus groups been used to develop a shared understanding of the drivers of the issue and of potential solutions to this issue? Has relevant software or resources been acquired, e.g., STICKE software, or paper-based resources.
	Implementation and evaluation planning	Have appropriate validated and reliable tools been identified and accessed to measure and assess change in the food environment?
		Are there defined roles and responsibilities for the stakeholders with regards to each action? Are there clear processes and implementation strategies for planning and implementing action? Is there commitment from key community leaders to support the action plan proposed? Have relevant implementation frameworks been considered?
	Implementation	Is there a clear method of prioritization for action?
	Feedback	Has progress been mapped to the ‘action plan’ to assess implementation progress? Has feedback to stakeholders/community been conducted and communicated? Have new barriers and enablers been identified? Is the key area of concern still your central focus?
Momentum continuous quality improvement cycle	Monitoring and evaluation	Have the post evaluation results been fed back to the governance group? Have the post evaluation results been communicated to stakeholders? Have the post-evaluation results been checked for policy alignment? Repeat appraisal (e.g., Store Scout App, Healthy diets ASAP tool Nutrition Environment Measure Survey https://nems-upenn.org/publications/)

COACH, CO-creation and evaluation of food environments to Advance Community Health.

### Stage 3: Coproduction

The prototype was presented to 2 online workshops that comprised 44 health promotion professionals and was separately presented to the author group and 4 independent stakeholders for feedback. An inductive thematic analysis of data collected through the workshops was conducted through multiple iterations of listening to audio recordings of the workshops. Direct quotes were transcribed to provide examples of specific points of improvement and needs for improved functionality of the prototype (Appendix File 3, available online, for the full thematic analysis with examples). For this group of participants, the major advantage of COACH was that it comprised all the important aspects of cocreation in one framework. One participant stated, “we have been working.... in an intuitive way[…] this is much needed.” Some elements were not clear such as the communication cycle as stated by a participant “…communications …. that [is] very, very important, … to ensure everybody [is] on board; … it could be more detailed about … how to support practitioners…”

Whereas the COACH checklist provided good guidance to follow the prototype and prompt discussions on where action was needed, participants provided various points of improvement for the prototype, including the need for case studies and examples. Participants provided ideas to improve and operationalize the prototype, for instance, “I would love if this is interactive enough to press on communication and then a tool came out, or I press on ‘evaluation’ and [a specific tool] came up.”

### Online Interactive Survey results

Over 60 participants registered for the 2 online workshops run in 2021; of these, 44 responded to a survey that requested feedback on the workshop. Thirty-one (70.5%) and 16% agreed and strongly agreed, respectively, that COACH was easy to interpret, with only 1 (2%) person disagreeing. Twenty-eight (79%) either agreed or strongly agreed that the checklist guided them through the COACH framework, 28 (76%) participants agree that they would use COACH in their work/research, 3 (9%) participants remained neutral, and 4 (12%) participants either disagreed or strongly disagreed. Full survey results are presented in Appendix File 3 (available online).

**Figure 3:**
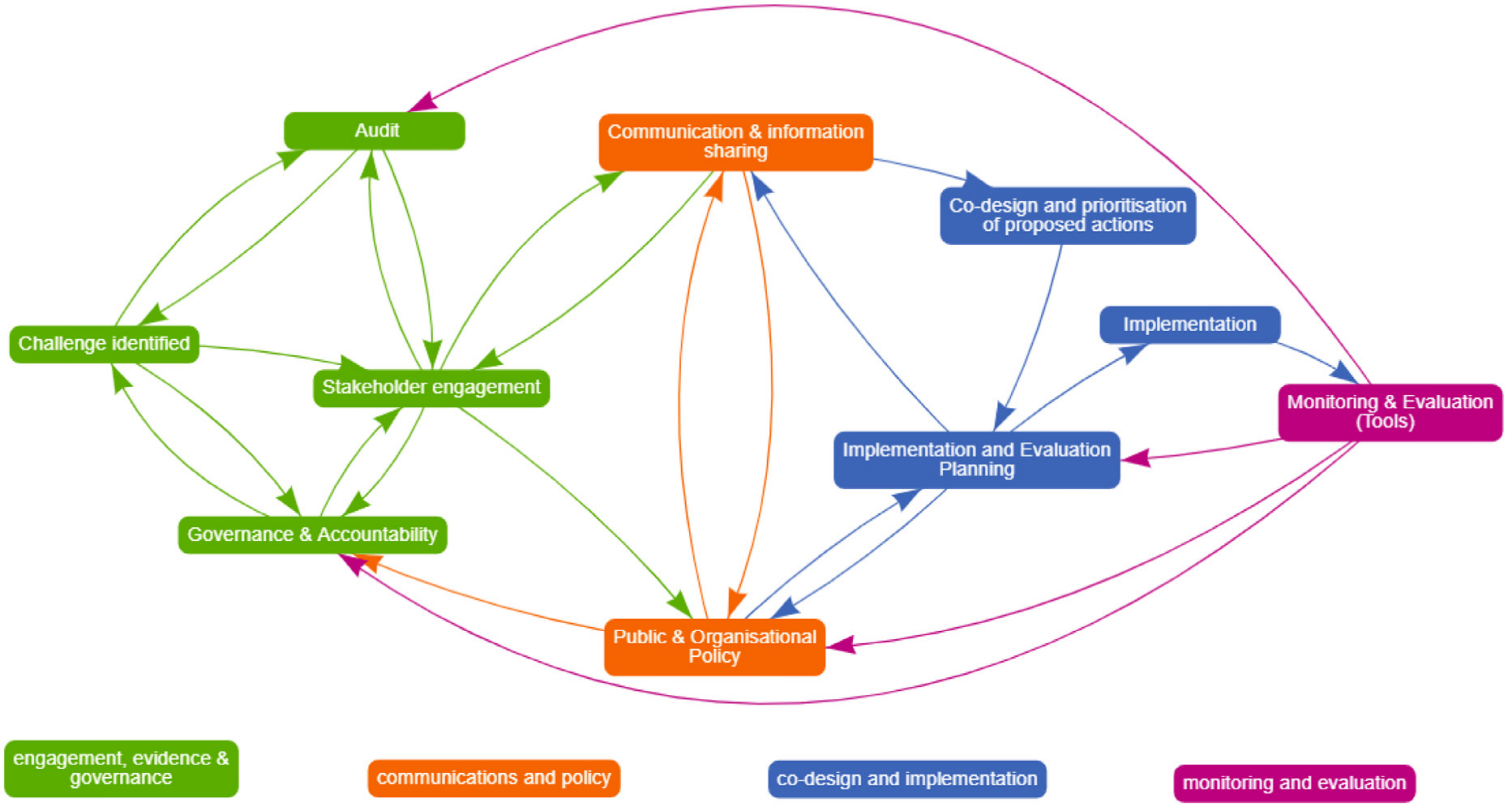
Framework: COACH. COACH, CO-creation and evaluation of food environments to Advance Community Health.

The prototype and the checklist were updated after consideration of all the feedback received both in real time and from the written evaluations of the workshops. This updated framework is presented in Figure 3. A summary of the different colored cycles will be described in the following sections.

#### GREEN: stakeholder engagement, evidence collection, and governance

The green cycle commences with the identification and understanding of the public health challenge to create food retail environments that promote health. Stakeholders include consumers; food retailers; community leaders; members of the food supply logistics chain; local, state, and federal government representatives; researchers; and policy advocates. There are very few validated and reliable tools for measuring and assessing the food retail environment.^37^ For evidence collection, which assesses the healthiness of the consumer food environment by providing informative baseline and outcome data, we have identified 2 electronic tools: the Store Scout App^38^ and Healthy Diets Australian Standardized Affordability and Pricing (ASAP) web-based portal.^39^ Store Scout can also be used to inform the codesign process because it provides evidence-informed best practice actions.^40^ The ASAP method compares the cost, cost differential, and affordability of current (unhealthy) and recommended (healthy, equitable, and more sustainable) diets in Australia.^41^ Healthy diets ASAP has been applied across a range of populations, including the whole of jurisdictional area,^42^ Aboriginal and Torres Strait Islander communities,^43^ rural communities,^39^ and cities.^44^

Within the green cycle, governance refers to the provision of coordination, guidance, impact, and vision for the work through consideration of responsibilities, interest and capacity, and policy pathway.^45^^,^^46^ Along with establishment of governance, there is merit in exploring its effectiveness, for example, the Good Food Systems project codesigned with stakeholders a capacity development appraisal tool to assess the effectiveness of the governance structure to effectively perform its task as a collective.^47^

#### ORANGE: communication and policy alignment/development

One of the most important aspects of communication at this stage is to ensure that all involved stakeholders receive good communication about progress and know their roles and responsibilities. To achieve this, a communication plan is developed that embraces multiple media opportunities to reach target audiences, for example, inclusion of user-friendly infographics.^48^

Where policies exist, stakeholders should ensure that these are communicated clearly before and during the codesign stage to ensure that prioritized actions align with the existing policy. If no current policies exist, opportunities may arise during this communication and policy alignment phase to consider whether new rules, regulations, or policies are required. For example, the Healthy Stores 2020 policy actions, a series of industry- and research-informed policy actions aimed at addressing merchandising practices, provide an example of existing policy guidance available for local store owners and retailers in remote Aboriginal and Torres Strait Islander communities in Australia for use when developing or reforming local policy.^40^

#### BLUE: codesign and implementation

The third phase uses participatory planning methods and/or systems methods to understand the drivers of the food environment and identify potential solutions. One process for participatory planning utilizing systems methods is Group Model Building (GMB).^49^ GMB is a tool from system dynamics^50^ that utilizes scripts to guide facilitated workshops.^51^ Along with aligned software, for example, STICKE,^52^ a shared understanding of a complex problem evolves and incorporates the diverse perspectives of multiple stakeholders. The process results in the development of a Causal Loop Diagram (CLD). Allender et al.^49^ have adapted the GMB process into 3 community workshops that culminate in action ideas, which are then prioritized for implementation, and implementation can be tracked onto the CLD.^53^ Prioritization factors include feasibility, community interest, energy, and momentum.^53^ Similar techniques have been used in remote Australia where stakeholders codesigned a food systems map with identified best practices (the Good Food Planning Tool) to be used to inform appraisal of different parts of a remote community food system and the planning and prioritizing of action for implementation. After the identification of prioritized actions in this step of codesign and implementation, an implementation plan is then devised utilizing an evidence-based planning tool that considers factors such as leadership, effectiveness, feasibility, and acceptability,^54^ followed by the roll out of implementation into the relevant food environment. Frameworks from the field of implementation science relevant to the implementation of health-enabling food retail initiatives include the Consolidated Framework for Implementation Research, where implementers are guided to consider contextual factors within the internal and external environments that may influence implementation of identified actions.^55^ To date, specific implementation guidance for food environment change has been limited. A framework specific to the implementation of health-enabling food retail environments is the Systems Thinking Approach for Retail Transformation (START) map, which identifies factors that assist with the uptake of existing healthier food retail initiatives.^56^ START identifies 17 implementation factors within 5 thematic areas that enable or impede the uptake of existing healthier food retail initiatives; these include changing retailer and consumer acceptance and challenges in accessing supply.^56^ Typically, successful implementation requires leadership, adequate resources, organizational commitment from key organizations, and the capacity to harness and advocate for resources to help facilitate action.^57^^,^^58^ These factors may prove useful in the planning for the implementation of the proposed actions that arise from COACH.

#### PINK: monitoring and evaluation

Tools used in baseline assessments are reapplied where needed in this phase to assess progress. CLDs created in Phase 4 provide a logic model to track implementation and guide evaluation.^59^ The Good Food Systems project used a participatory Ripple Tool to appraise and monitor performance in best practice action areas of the food system with time^47^ and used food retail point of sale data to provide reports on the performance of key indicators.^60^

### The COACH Checklist for Implementation

Within Step 2 of the prototype development to assist with implementing the COACH framework, a checklist was developed (Table 3). It is not necessary to work through each phase sequentially. For example, where a government policy or a governance structure exists, starting with evidence collection in Phase 1 and/or Phase 2 may be most appropriate. However, to create a fully formed initiative that incorporates continuous quality improvement processes, all phases should be considered.

## DISCUSSION

A 3-stage multimethod process for the coproduction and prototyping of public health interventions was used to create the COACH framework. COACH is the first framework to describe what best practice application of cocreation in health-enabling food retail environments should involve. COACH is informed by an evidence review and consultation with health promotion practitioners, stakeholders, and key experts in the field. The framework comprises 4 key phases: (1) engagement, evidence, and governance; (2) communication and policy alignment; (3) codesign and implementation; and (4) monitoring and evaluation. The cyclical nature of COACH also emphasizes the need for continuous quality improvement.

COACH strengthens existing cocreation frameworks; for example, the broad checklist proposed by Leask et al.^22^ provides useful guidance to cocreation at a point in time, but given the complexity of the food environment, we propose the COACH multicycle iterative framework, and guiding checklist assists to address the complexity of the food environment and the need to engage multiple stakeholders at multiple time points. It is in this way that COACH envisages cocreation as a continuum that regularly checks in with stakeholders relevant to each stage and incorporates continuous quality improvement cycles.

COACH adopts a systems perspective to cocreating health-enabling food retail and approach that aligns with all the 10 key factors of a whole systems approach identified by Bagnall et al.^61^ COACH is designed to build capacity in both researchers and practitioners and guide change making in food environments. COACH requires clear and consistent stakeholder commitment and alignment with policy, resourcing, and sustained high-level leadership and management support. Along with addressing system change factors, COACH addresses elements essential for collaborative problem solving.^45^^,^^46^^,^^57^^,^^62^

Integral to working through the proposed phases of COACH is the importance of building trust, policy, and support for community health as factors that influence food store owners and managers to change practice and aligns with previous work.^63^ Our previous research in remote Aboriginal community settings in Australia found that local multisector groups valued the leadership of local community people, the input of a facilitator to guide progression through the continuous quality improvement steps, and the provision of evidence.^45^ Successful implementation of actions prioritized by the community groups was dependent on a number of factors, including high-level leadership and commitment to implementation from the relevant organizations represented in the community and the capacity of the group to harness and/or advocate for resources to help facilitate action.^45^^,^^57^^,^^64^

COACH brings together multiple concepts with specific reference to the cocreation of healthy food retail, including trust, healthy policy, and community health to support food store owners.^63^ This framework builds on concepts of adaptability and resilience in food retail^65^ and the START map, which identifies factors that assist with the uptake of existing health-enabling food retail initiatives.^56^ COACH is informed by evidence developed in unique contexts; for example, research with Aboriginal communities in remote Australia found that local community leadership, facilitation through the continuous quality improvement steps, inclusion of evidence, high-level leadership, capacity to harness resources, and commitment of community organizations to implementation^45^^,^^57^ were critical to success and sustained action.^64^

### Limitations

COACH provides a process framework that can be utilized across multiple types of food retail initiatives. It is iterative and adaptive rather than prescriptive. It is founded in practice and research and drawn from successful community programs. COACH was developed within the first international CRE in food retail environments to promote health. The authors of COACH drew on the evidence and experience of these leading international experts in the field. A rigorous methodology was followed in the development and testing of the face validity of COACH.

Although the systematic review collated international evidence from authors within and external to the CRE, it is possible that some cocreation expertise reported in gray literature was missed. We consider this impact to be minimal because the authors considered gray literature during preparation for the workshops in the development of the prototype. Most data and tools have been derived from high-income countries; COACH should be tested in low- and middle-income countries for face validity. We consider that COACH is likely relevant to these countries because much of this work that informed COACH has been tested in low-resourced communities and settings within Australia.

## CONCLUSIONS

COACH represents a comprehensive, adaptive, iterative process framework to guide the development of inclusive and equitable health-enabling food environments. This paper adds an evidence-informed framework for working with communities to improve the healthiness of food retail environments. The framework has been designed to empower communities to be proactive in creating health-enabling food retail environments.

Future research will pilot test the use of COACH in a variety of food retail settings to assess the feasibility and acceptability of the framework. These will be measured through interviews with multiple stakeholders involved in the cocreation process.

Future research could also test the systems impact of the cocreated actions through the application of the Action Scales Model^66^ and/or Public Health 12 Framework.^67^ These frameworks assess where specific actions meet leverage points for change in the system to provide insights on the most effective places to intervene.

## Appendix Supplementary materials

Image, application 1

Image, application 2

Image, application 3

## Footnotes

Supplementary material associated with this article can be found in the online version at doi:10.1016/j.focus.2023.100111.
