# Supplementary material for: CO-Creation and Evaluation of Food Environments to Advance Community Health (COACH)
Source: AJPM Focus. 2023 May 27;2(3):100111. doi: 10.1016/j.focus.2023.100111 (PMC10546519; doi:10.1016/j.focus.2023.100111)
Supplement: Supplementary file 1 [file mmc1.docx]

**Appendix File 1**

**Key papers used to guide the development of the CO-creation and evaluation of food environments to Advance Community Health (COACH) framework**

| **Authors (Year) title** | **Stakeholder engagement, evidence collection and governance** | **Communication, policy alignment and development** | **Community engagement and co-design of evidence-informed action** | **Implementation** | **Feedback and evaluation** | **Momentum continuous quality improvement cycle** |
| --- | --- | --- | --- | --- | --- | --- |
| Allender et al. (2021)^64^ | 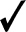 |  | 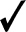 | 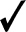 | 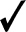 | 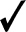 |
| Allender et al. (2019)^58^ | 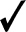 |  | 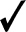 | 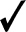 | 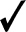 | 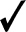 |
| Allender et al. (2016)^65^ | 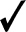 |  | 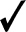 | 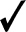 | 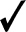 |  |
| Allender et al (2015)^47^ | 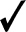 |  | 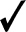 |  |  |  |
| Alston et al (2021)^14^ |  |  |  |  | 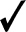 |  |
| Alston et al (2020) ^15^ |  |  |  |  | 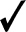 |  |
| Boelsen-Robinson et al. (2020)^53^ |  |  |  | 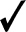 |  |  |
| Brimblecombe et al. (2020)^22^ | 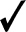 | 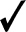 | 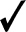 | 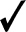 | 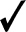 | 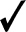 |
| Brimblecombe et al. (2015)^26^* | 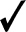 | 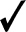 | 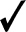 | 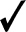 | 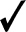 | 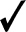 |
| Brimblecombe et al (2017)^27^* | 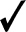 | 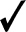 | 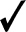 | 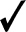 | 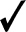 | 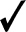 |
| Brimblecombe et al. (2014)^45^* | 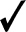 | 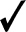 | 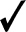 | 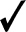 | 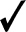 | 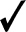 |
| Ferguson & Brimblecombe (2021)^28^ |  | 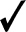 | 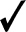 |  |  |  |
| Jaenka et al. (2021)^66^ | 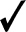 |  |  |  |  |  |
| Lee et al. (2020)^43^ | 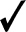 | 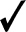 |  | 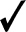 | 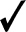 |  |
| Lee et al (2018)^40^ | 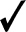 | 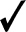 |  | 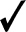 | 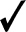 |  |
| Lee et al (2013)^67^ | 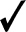 | 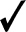 |  | 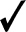 | 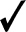 |  |
| Lee et al (2021)^41^ | 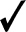 | 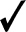 |  | 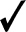 | 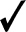 |  |
| Maitland (2021)^51^ | 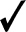 | 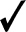 | 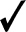 | 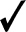 | 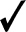 | 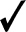 |
| Mc Mahon et al (2020)^39^ | 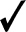 |  |  |  |  |  |
| Rogers et al (2018)^54^* | 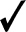 | 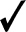 | 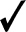 |  | 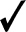 |  |
| Sweeney et al (2018)^68^ | 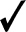 |  | 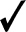 | 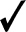 | 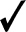 |  |
| Savona et al (2021)^69^ | 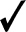 | 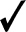 | 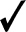 | 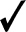 | 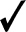 | 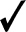 |
| Love et al. (2018)^40^ | 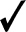 | 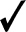 |  |  | 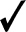 |  |
| Whelan et al. (2021)^62^ | 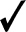 |  |  |  | 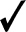 |  |
| Whelan et al. (2018)^13^ | 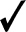 | 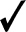 | 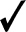 | 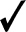 | 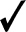 | 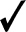 |
| *From the good food system research trial^26^ | | |  |  |  |  |
