# Supplementary material for: CO-Creation and Evaluation of Food Environments to Advance Community Health (COACH)
Source: AJPM Focus. 2023 May 27;2(3):100111. doi: 10.1016/j.focus.2023.100111 (PMC10546519; doi:10.1016/j.focus.2023.100111)
Supplement: Supplementary file 2 [file mmc2.docx]

# **Appendix File 2 Workshop outline:**

|  | **Order** | **Time** |
| --- | --- | --- |
| 1 | **Open and welcome**   - Housekeeping and gathering of PLS and consent | 10 minutes |
| 2 | **Introduce COACH – why have we design and what is the COACH Framework (approx. 10-minutes)**   - 1. Description of COACH   2. E-tools data collection   3. Group Model building   4. Example | 40 minutes |
| 3 | **Break** | **5 minutes** |
| 4 | **COACHVille (video)** | 5 minutes |
| 5 | **Group activity 1**  Explore how their group would you improve one of the “COACHVILLE” food environments using the checklist as a guide to support them through this process.  For the first 15 minutes please address the following questions to guide the discussion:  **Discussion guiding questions:**   1. What scenario did you choose? 2. What is the challenge that you are trying to approach? 3. What stakeholders need to be involved? 4. What COACH cycle are you using as a starting point? Why? 5. How would you progress from this COACH cycle?   **For the second half (15minutes) please ask the following questions**  The following questions will be asked:  Did you find the COACH framework easy to apply to the scenario?   - What did you like? - What did you not like? - Would you use the COACH framework in your current position and if so, please provide an example?   **Group Sharing once back from the breakout rooms**   - Discuss key points key points from each group (either note taker or participant) | 30 minutes |
| 6 | **Break** | 15 minutes |
| 7 | Short evaluation survey  Did you find the COACH framework to easy to interpret?   - Where did you start in the COACH framework? - What phase was most difficult to work through? (multi choice) - What did you like? (word clouds) - What did you not like? (word clouds) - Did the checklist guide you through the COACH framework? (Likert) - What did you think was missing from the COACH framework? (word cloud) - Do you think you would use COACH in your work/research? Yes, No, Maybe - What word would you use to describe COACH? (word clouds) - Are there any other comments you would like to make relating to the COACH framework? Open ended… | 15 minutes |
| 8 | **Group Activity 2**  COACH Checklist   - Did the Checklist guide you through the process? - How can we improve the check list? | 15 minutes |
| 9 | Closing summary | 5 minutes |
