# Supplementary material for: CO-Creation and Evaluation of Food Environments to Advance Community Health (COACH)
Source: AJPM Focus. 2023 May 27;2(3):100111. doi: 10.1016/j.focus.2023.100111 (PMC10546519; doi:10.1016/j.focus.2023.100111)
Supplement: Supplementary file 3 [file mmc3.docx]

**Appendix File 3.** *Online survey results from stakeholder workshops run in October and November 2021*

| **ONLINE QUESTIONS** | **Strongly agree** | **Agree** | **Neutral** | **Disagree** | **Strongly disagree** |  | **Total Response per question** |
| --- | --- | --- | --- | --- | --- | --- | --- |
| **Did you find the COACH framework easy to interpret?** | 7 (16%) | 31 (70.5 %) | 5 (11.5%) | 1 (2%) | 0 |  | 44 |
| **Did the checklist guide you through the COACH framework?** | 9 (25 %) | 19 (54 %) | 3 (9 %) | 3 (9 %) | 1 (3 %) |  | 35 |
|  | **Stakeholders' engagement & governance** | **Communication and policy alignment** | **Community engagement and co-design-informed action** | **Implementation** | **Feedback and evaluation** | **Momentum continuous quality improvement cycle** |  |
| **Where did you start in the COACH framework** | 39 (90.5%) | 3 (7 %) |  | 1 (2%) |  |  | 43 |
| **What phases was the most difficult to work through?** | 6 (21 %) | 7 (24 %) | 7 (24 %) | 6 (21 %) | 1 (3.5 %) | 2 (7 %) | 29 |
|  | **Yes** | **Maybe** | **No** |  |  |  |  |
| **Do you think you would use COACH in your work/research?** | 28 (76 %) Yes | 9 (24 %) | 0 (0%) |  |  |  | 31 |
